# Influence of Oxygen on Chemoconvective Patterns in the Iodine Clock Reaction

**DOI:** 10.1021/acs.jpcb.2c04682

**Published:** 2022-11-23

**Authors:** Haimiao Liu, Annette F. Taylor

**Affiliations:** † School of Chemical Engineering and Technology, China University of Mining and Technology, Xuzhou221116, China; ‡ Chemical and Biological Engineering, University of Sheffield, SheffieldS1 3JD, U.K.

## Abstract

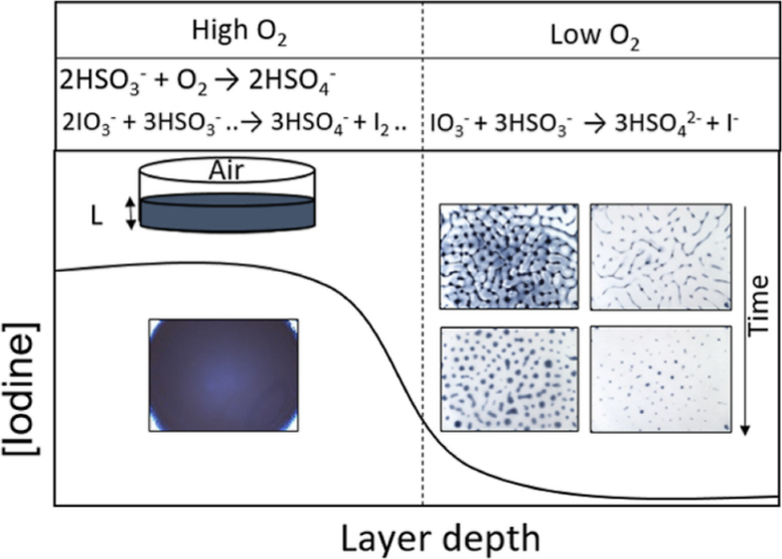

There is increasing interest in using chemical clock reactions to drive material formation; however, these reactions are often subject to chemoconvective effects, and control of such systems remains challenging. Here, we show how the transfer of oxygen at the air–water interface plays a crucial role in the spatiotemporal behavior of the iodine clock reaction with sulfite. A kinetic model was developed to demonstrate how the reaction of oxygen with sulfite can control a switch from a low-iodine to high-iodine state under well-stirred conditions and drive the formation of transient iodine gradients in unstirred solutions. In experiments in thin layers with optimal depths, the reaction couples with convective instability at the air–water interface forming an extended network-like structure of iodine at the surface that develops into a spotted pattern at the base of the layer. Thus, oxygen drives the spatial separation of iodine states essential for patterns in this system and may influence pattern selection in other clock reaction systems with sulfite.

## Introduction

Many biological, geological, and engineering systems show structure or pattern formation driven by the coupling of chemical reaction with convection. Reactions are able to trigger hydrodynamic flows by changing the physical properties of the fluid, such as its density, viscosity, or surface tension. These chemoconvective (or chemohydrodynamic) flows, in turn, can affect the spatiotemporal dynamics of the system resulting in oscillations in concentrations, formation of cellular structures, or fingering instabilities.^1^ Examples include chemically controlled contraction waves in the slime mold,^2^ orange peel effect in drying paint films,^3^ and pulsating instabilities in frontal polymerization for the manufacture of composites.^4,5^ Unravelling the mechanism of pattern formation in such systems has been frequently hindered by the complexity associated with multiple processes taking place simultaneously, and it remains challenging to control chemoconvective patterns.^6^

An important class of self-organized structures is found in autocatalytic systems far from equilibrium.^7^ Such systems show clocks under stirred conditions (large amplitude changes in the product after an induction period) and reactive interfaces propagating with constant velocity in unstirred solutions.^8^ Reaction-diffusion fronts or waves in such systems can be deformed and accelerated because of the gradients in density, viscosity, and/or surface tension that result from the chemical reactions.^9−11^ Examples include the hydrodynamic flows associated with chemical wave propagation in the classical Belousov–Zhabotinsky system,^12,13^ buoyancy-induced convection of fronts in the chlorite–tetrathionate reaction,^14^ and viscous fingering in the formaldehyde-sulfite reaction.^15^ A well-studied example system is the iodine clock reaction, first discovered by Landolt in 1886,^16^ which involves iodate oxidation of sulfite or other reductants including arsenous acid and is autocatalytic in both protons and iodide ions.^17^ This reaction has displayed fingering attributed to double-diffusive instabilities,^18^ Marangoni effects, and density fingering.^19^ Nevertheless, there is increasing interest in the use of autocatalytic reactions for the spatiotemporal programming of material formation, for example, in the coupling of the iodine clock reaction with poly(vinyl alcohol) gelation, the formaldehyde-sulfite or chlorite–tetrathionate reaction with pH-sensitive self-assembly, or the urea–urease reaction with thiol-acrylate polymerization.^20−25^

A rich variety of convective patterns can also arise from relatively simple chemical reactions taking place at interfaces. The line and dot patterns in the methylene-blue-glucose-oxygen “blue-bottle” system^26^ and enzymatic “green-bottle”^27^ occur from a Rayleigh-Taylor or overturn the instability caused by formation of dense products from reactions with oxygen at the air–solution interface. Buoyancy-driven convection also plays an important role in the synthesis of complex precipitation structures, or chemical gardens, in the growing field of chemobrionics.^28^ Surface tension-driven flows from chemical gradients at liquid–liquid or air–liquid interfaces lead to Marangoni instabilities and associated patterns.^29−31^ The spot and labyrinth patterns obtained in some photochemical reactions at the air–water interface was later attributed to the presence of a prepattern caused by evaporation at the air–water interface; in this case, the chemical product traced the existing pattern rather than driving its formation.^32,33^

Much of the experimental work on fronts in the Landolt reaction has been performed in quasi-two-dimensional (2D) configurations, for example, Hele-Shaw cell or a continuous fed unstirred reactor, and the effect of convection at the air–water interface on three-dimensional (3D) pattern formation has not been elucidated.^34−36^ Here, we found that the clock reaction can display spatiotemporal patterns in a thin layer open to air, and we show that oxygen plays a crucial role in pattern development by controlling the amount of iodine formed. The stoichiometric ratio *S* = [SO_3_^2–^]_0_/[IO_3_^–^]_0_ is an important parameter in the Landolt clock reaction where for *S* < 3, iodine is formed along with the blue starch–triiodide complex, but for *S* > 3, iodide ion is in excess and no color change is observed. Kinetic simulations were used to show that oxygen removes bisulfite resulting in an effective decrease in *S* and drives the formation of gradients in iodine for appropriate initial values of *S*. With a fixed value of *S* = 3.5, these iodine gradients combined with convective effects to give rise to spotted iodine patterns at optimal layer depths. This work shows how oxygen may be used to influence chemoconvective pattern selection in clock reactions involving sulfite, important for future applications of such systems.

## Experimental Methods

Stock solutions of starch (6.154 g/L), KIO_3_ (0.144 M), Na_2_SO_3_, (0.499 M), and H_2_SO_4_ (0.0768 M) were prepared from analytical grade reagents (Sigma-Aldrich) and deionized water. Each experiment was initiated by mixing stock solutions of starch, sulfuric acid, sulfite, and iodate in that order to obtain solutions with different initial concentrations and total volume of 40 mL after mixing. Sulfuric acid reacts with sulfite to form bisulfite and a SO_3_^2–^/HSO_3_^–^ buffer. The initial concentrations of potassium iodate [KIO_3_]_0_ and sulfuric acid [H_2_SO_4_]_0_ were fixed at 8.96 and 4.80 mM, respectively. Here, []_0_ denotes the concentration of the added species after mixing and prior to any reaction.

The homogeneous kinetics experiment was conducted in a thermostated glass vessel at 27 ± 0.2 °C equipped with a magnetic stirrer rotating at 800 rpm to ensure uniform mixing. The pH signal in the vessel was monitored with a glass electrode coupled to an Hg|Hg_2_SO_4_|K_2_SO_4_ reference electrode, and the data were collected by a computer equipped with an e-corder/201 (eDAQ) system.

Propagating fronts and pulses of iodine were obtained under nonstirred conditions in a glass Petri dish of diameter 48 mm, illuminated by a MiniSun A4 light-emitting diode (LED) light pad from below. The solution was prepared as detailed above, and one drop of Triton X-100 was added to facilitate spreading in the Petri dish. Then, 1.8 mL of solution was pipetted into the Petri dish to give a layer depth of 0.9 mm. Experiments were performed in duplicate. The reaction was initiated by addition of a drop of product solution (10 μL) from the reaction with sulfite = 0.027 M; initiation could also be achieved with sulfuric acid or potassium iodide. Images of the Petri dish were taken at 10 s intervals with a digital camera (PixeLink) mounted vertically above the Petri dish. A microscope slide glass coverslip (22 × 22 × 0.17 mm) was placed carefully at the air–water interface in some experiments.

For the observation of iodine gradients perpendicular to the air–water interface, a side view of the unstirred reaction was obtained in a thin glass container of dimensions 5 × 22 × 34 mm. Iodine appeared spontaneously after an induction period, and the reaction was followed with a digital camera (PixelLink) mounted horizontally. Illumination with a MiniSun A4 LED light pad was used. In experiments under nitrogen in place of air, the solution was bubbled with nitrogen for 1 min, and then the container was filled with nitrogen and sealed with a rubber stopper.

All experiments on network/spotted pattern formation were conducted in a Petri dish with a diameter of 88 mm; a thin solution layer in the Petri dish had a free surface or was loosely covered with a lid. The solution was prepared as described above. Then, we poured an appropriate amount of the mixed solution into the Petri dish and spread it to create a thin solution layer with a constant thickness. The patterns were spontaneously initiated after a period of time by perturbations such as the concentration gradient of reactants or products. The evolving patterns were monitored using a digital camera (PixeLINK, PL-B952U). The camera was mounted directly above the Petri dish or at an angle of 30° to observe the patterns throughout the layer depth. The length scale was determined from reference images with mm graph paper. The system was illuminated from below by a MiniSun A4 LED light pad, and images were captured with a fixed time interval in one experiment. In order to determine any influence of oxygen, a microscope slide glass coverslip (22 × 22 × 0.17 mm) or a 47 mm filter paper (Millipore, 0.45 μm pore size) was placed carefully at the air–water interface or the Petri dish containing reaction solution was placed in a vacuum desiccator which was purged with nitrogen.

Shadowgraphs and transmission images of structures in reaction solution or starch only solution in an 88 mm diameter Petri dish were taken with a digital camera (PixeLink). A white LED (diameter: 5 mm, epoxy dome removed) was used for light source with a 100 mm diameter and a 200 mm focal length plano convex condenser lens (Edmund Optics) placed in the light path just underneath the dish holder stage.

Images were analyzed using a combination of ImageJ (for space-time images) and Matlab (for front position). The average position of the front and standard deviation was determined from of nine equidistantly spaced intensity profiles (green channel of RGB image) taken from the center point of the initiation to the edge of the Petri dish. Fronts were allowed to develop to a radius of 3 mm before measurements were taken. The peak in the derivate of the intensity versus distance intensity profiles marked the position of the front in an experiment. The front velocity was determined from the slope of the plot of average front position in time. The linear portion of the position-time plot was used in the case of lower *S*, where acceleration of the font was observed at later times. For the experiments viewed from the side, the average intensity was obtained from the average intensity of a rectangular section of the image (encompassing 17 × 2 mm of the reactor) placed directly below the meniscus. The final intensity was determined at the end of the experiment, after formation of iodine, when the average intensity did not change over a time period of at least 50 s. The reaction time was obtained from the time to the minimum in the average intensity-time plot, which corresponded to the maximum amount of iodine (dark). The space-time plot was constructed from a slice of the image from the bottom of the container up to the meniscus. Absorbance profiles [log(*I*/*I*_0_)] were constructed by extracting the intensity along a profile (line indicated on image) at the start of the experiment to obtain *I*_0_ as a function of distance (with the left end of the profile designated as *x* = 0) and extracting the intensity along the same profile to obtain *I* at a later time.

The experiments on fronts and pattern formation reported here were conducted at room temperature (22 ± 2 °C). Some additional experiments were also performed at a higher temperature of 27 ± 2 °C, and qualitatively similar patterns were obtained with changes in sulfite and layer depth; however, the clock reaction time was faster. Temperature did not appear to influence the switch between the low-iodine and high-iodine states reported here.

### Model

The model for the iodine clock reaction was taken from earlier work with rate constants primarily determined at 25 °C.^17^ The aim of the model was to qualitatively reproduce the effect of oxygen on the reaction, with some rate constants adjusted to obtain a reasonable match to the well-stirred experiments. Three overall processes were included; first, the slow oxidation of bisulfite
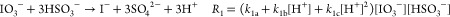
1with rate law *R*_1_ where *k*_1a_ = 0.08 M^–1^ s^–1^, *k*_1b_ = 4 × 10^3^ M^–2^ s^–1^, and *k*_1c_ = 3 × 10^5^ M^–3^ s^–1^. Iodine was formed by oxidation of iodide in (eq 2), the Dushman reaction, and removed by reaction with bisulfite in (eq 3)
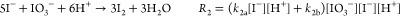
2
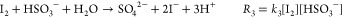
3with *k*_2a_ = 2.2 × 10^9^ M^–4^ s^–1^, *k*_2b_ = 25 M^–2^ s^–1^, and *k*_3_ = 2 × 10^9^ M^–1^ s^–1^. The following reversible reactions governed the pH^37^
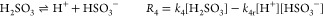
4
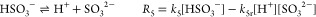
5
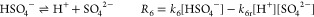
6
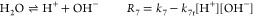
7where *k*_4_ = 2.6 × 10^6^ s^–1^, *k*_4r_ = 2 × 10^8^ M^–1^ s^–1^, *k*_5_ = 5 × 10^3^ s^–1^, *k*_5r_ = 5 × 10^10^ M^–1^ s^–1^, *k*_6_ = 1.3 × 10^8^ s^–1^, *k*_6r_ = 1 × 10^10^ M^–1^ s^–1^, *k*_7_ = 1 × 10^–3^ M s^–1^, and *k*_7r_ = 1 × 10^11^ M^–1^ s^–1^. The tri-iodide equilibrium reaction provided the I_3_^–^ necessary for formation of the starch–triiodide complex, which acts as a color indicator in experiments^38^
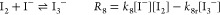
8with *k*_8_ = 5.6 × 10^9^ M^–1^ s^–1^ and *k*_8r_ = 8.5 × 10^6^ s^–1^. The reaction of sulfite or bisulfite with oxygen is complex and involves radical species.^39^ Various simple rate equations have been suggested that only apply under certain sets of conditions. Here, the following overall reaction was included for the process^40^
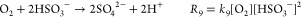
9

The rate constant was taken as *k*_9_ = 1500 M^–1^ s^–1^. Under well-stirred conditions, the transfer from the gas to the solution was accounted for by
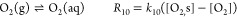
10where [O_2,_s] is the saturated solution concentration and [O_2_] is the concentration in solution. The mass transfer rate *k*_10_ depends on numerous factors including stirring rate and is of the order of 1 × 10^–4^ to 1 × 10^–2^ s^–1^ for well-stirred tank reactors.^41^ Here, the value was taken as 5 × 10^–3^ s^–1^ to best match the experimental results. The saturated solution concentration of oxygen, [O_2,_s] = 2.56 × 10^–4^ M, was taken from Henry’s law constant at *T* = 25 °C:^42^*H* = [O_2,s_]/*p*_o2_ where *H* = 1.2 × 10^–3^ mol dm^–3^ atm^–1^ and *p*_o2_ = 0.21 atm. The equations were solved using XPP-AUT with integration method cvode.

In order to illustrate the influence of oxygen on the amount of iodine formed under nonstirred conditions (without pattern formation), the reaction was also modeled in a one-dimensional (1D) vertical slice, with transfer of oxygen included from the gas phase to the liquid and mass transport of all species in solution through diffusion. The rate of change of the ith species is given by
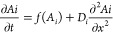
11where *f*(A_*i*_) contains reaction terms from reactions 1–10 in the mechanism given above and *D*_*i*_ is the diffusion coefficient of the *i*th species. The diffusion coefficient of species was 1 × 10^–3^ mm^2^ s^–1^ except for H^+^ which was 9 × 10^–3^ mm^2^ s^–1^ and for OH^–^ which was 5 × 10^–3^ mm^2^ s^–1^.^37^ The diffusion coefficient of O_2_ was 2 × 10^–3^ mm^2^ s^–1^.^43^ The reaction was modeled using XPP-AUT with integration method cvode and method of lines for space with 75–150 grid points and spatial step size 0.02 mm. The boundary conditions were no flux at *x* = 0 and *x* = L, except for oxygen which at *x* = 0 is given by
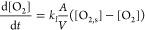
12where *k*_l_ is the liquid-side mass-transfer coefficient for oxygen, *A*/*V* is the ratio of gas–liquid surface area to volume of liquid, and [O_2,_s] = 2.5 × 10^–4^ M is the saturation concentration of oxygen. The value of *k*_l_ was taken as 0.1 mm s^–1^ and the *A*/*V* was determined for a rectangular container as 1/*d* where *d* is the solution depth. The initial conditions were [IO_3_^–^] = 8.96 mM, [H^+^] = 4.8 mM, [HSO_4_^–^] = 4.8 mM, and [SO_3_^2–^] = 0.024 M and all other species set to zero, unless otherwise stated.

## Results

**Figure 1:**
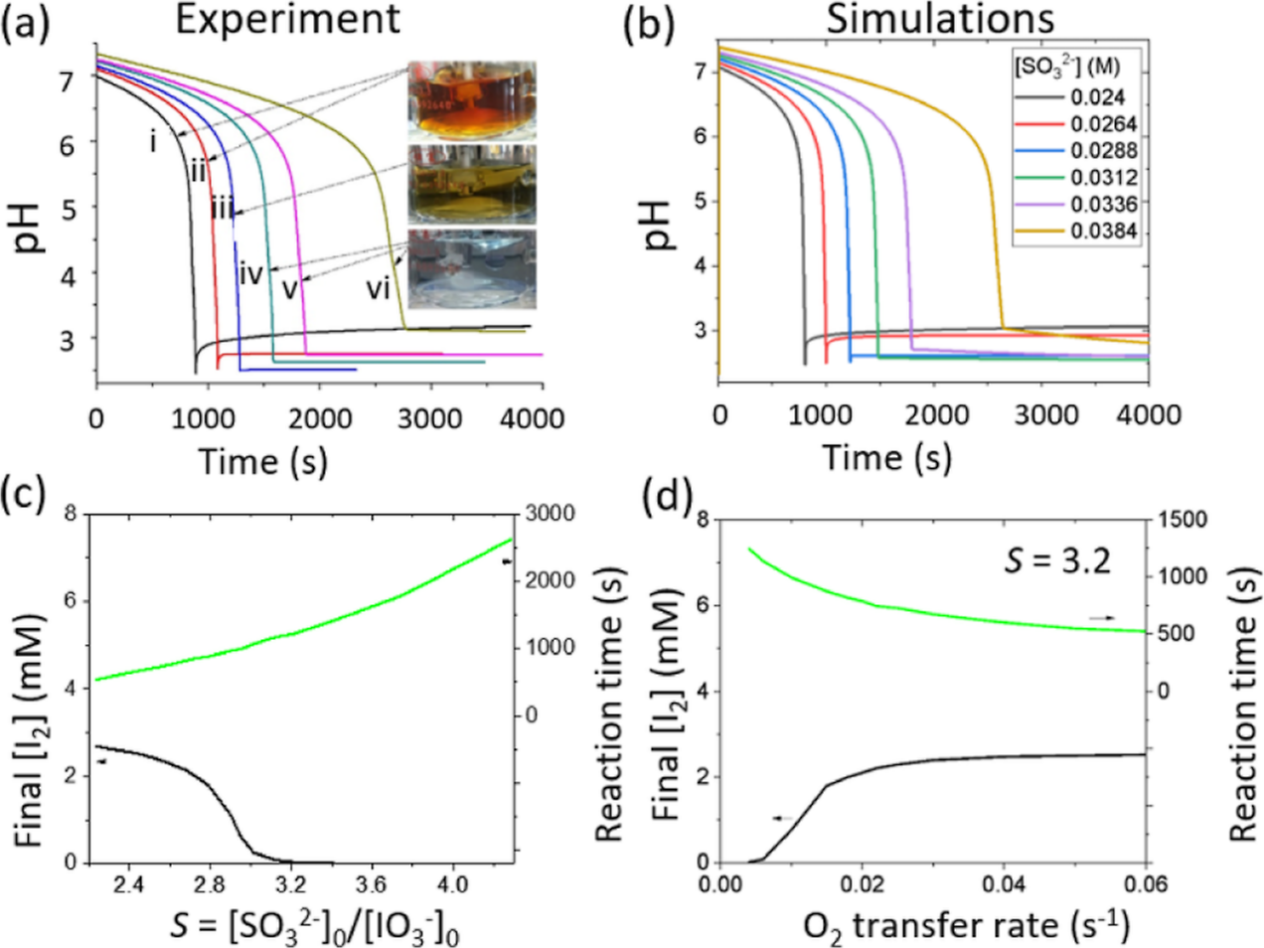
Experiment (a) and simulations (b–d) of pH versus time for the IS reaction in a well-stirred batch reactor with different initial concentrations of sulfite and increasing stoichiometric ratio, *S* = [SO_3_^2–^]_0_/[IO_3_^–^]_0_: [Na_2_SO_3_]_0_ = 2.40 × 10^–2^ mol/L (i), 2.64 × 10^–2^ mol/L (ii), 2.88 × 10^–2^ mol/L (iii), 3.12 × 10^–2^ mol/L (iv); 3.36 × 10^–2^ mol/L (v), and 3.84 × 10^–2^ mol/L (f). (c) Final concentration of iodine (*T* = 4000 s) as a function of *S* and reaction time to pH 4 (green, upper). (d) Final concentration of iodine as a function of O_2_ transfer rate (*k*_10_) and reaction time to pH 4 (green, upper). Other fixed conditions: [KIO_3_]_0_ = 8.96 × 10^–3^ mol/L, [H_2_SO_4_]_0_ = 4.80 × 10^–3^ mol/L. Influence of oxygen on iodine fronts and pulses.

The iodate–sulfite (IS) clock reaction is autocatalytic both in protons and iodide ions.^44,45^ The fast reversible sulfite–bisulfite equilibrium reaction (*R*_5_) has a buffering effect, which keeps the system at a relatively high pH and the bisulfite constant with [HSO_3_^–^] = *K*_a_[H^+^][SO_3_^2–^]. After a well-defined induction period or clock time, the dominant positive feedback process involving protons occurs rapidly (*R*_3_) resulting in a pH drop, as illustrated in Figure 1a. The iodide autocatalytic process (*R*_2_ + 3*R*_3_: IO_3_^–^ +3HSO_3_^–^ + 5I^–^ + 6H^+^ → 3SO_4_^2–^ + 6I^–^ + 9H^+^) becomes important at the end of the sulfite oxidation process.

The reaction time is defined as the time for the pH to drop to 4, and it increases with increasing concentration of sulfite as it takes longer for the buffer to be consumed. The outcome of the reaction (low or high iodine) in the IS reaction depends on the stoichiometric ratio
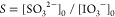
13

When the stoichiometric constraint *S* < 3 was met, there was a sudden color change after complete consumption of bisulfite and the main product was iodine.
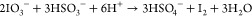
I

The pH dropped to a minimum and then increased through *R*_2_, as shown in Figure 1a(i) (*S* = 2.7). When *S* was ∼3, a mixture of iodine and iodide formed [Figure 1a(ii), *S* = 2.95]. When the initial sulfite concentration was increased so that *S* > 3, iodine was completely consumed in *R*_3_ and the main product was iodide [Figure 1a(iii,iv), *S* = 3.2–4.3]
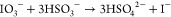
II

Simulations of reactions 1–10 reproduced the increase in the reaction time and the switch from high- to low-iodine state with increasing *S* obtained through increasing sulfite concentration (Figure 1b,c). The model can also be used to explore the effect of oxygen on the reaction. An increase in the transfer rate of oxygen (through *k*_10_) resulted in a decrease in the reaction time and transition from the low- to high-iodine state because oxygen consumed bisulfite (and hence sulfite) through *R*_9_ in an alternative reaction pathway (Figure 1d). Increasing oxygen has the same effect as decreasing sulfite or *S*, with iodine as the major product instead of iodide.

The IS reaction has a mechanism similar to the iodate–arsenous acid (IA) reaction with arsenous acid as the reductant in place of sulfite; the IA reaction is also autocatalytic in both acid and iodide, and the behavior depends on the stoichiometric ratio of the reductant to iodate: *R* = [H_3_AsO_3_]_0_/[IO_3_^–^]_0_.^46,47^ In thin layers in a Petri dish (0.8 mm depth), when *R* < 3, the IA reaction supports propagating fronts of iodine, whereas for higher arsenous acid concentrations, *R* ∼ 3, a narrow band or pulse of iodine may be obtained.^46,48^

**Figure 2:**
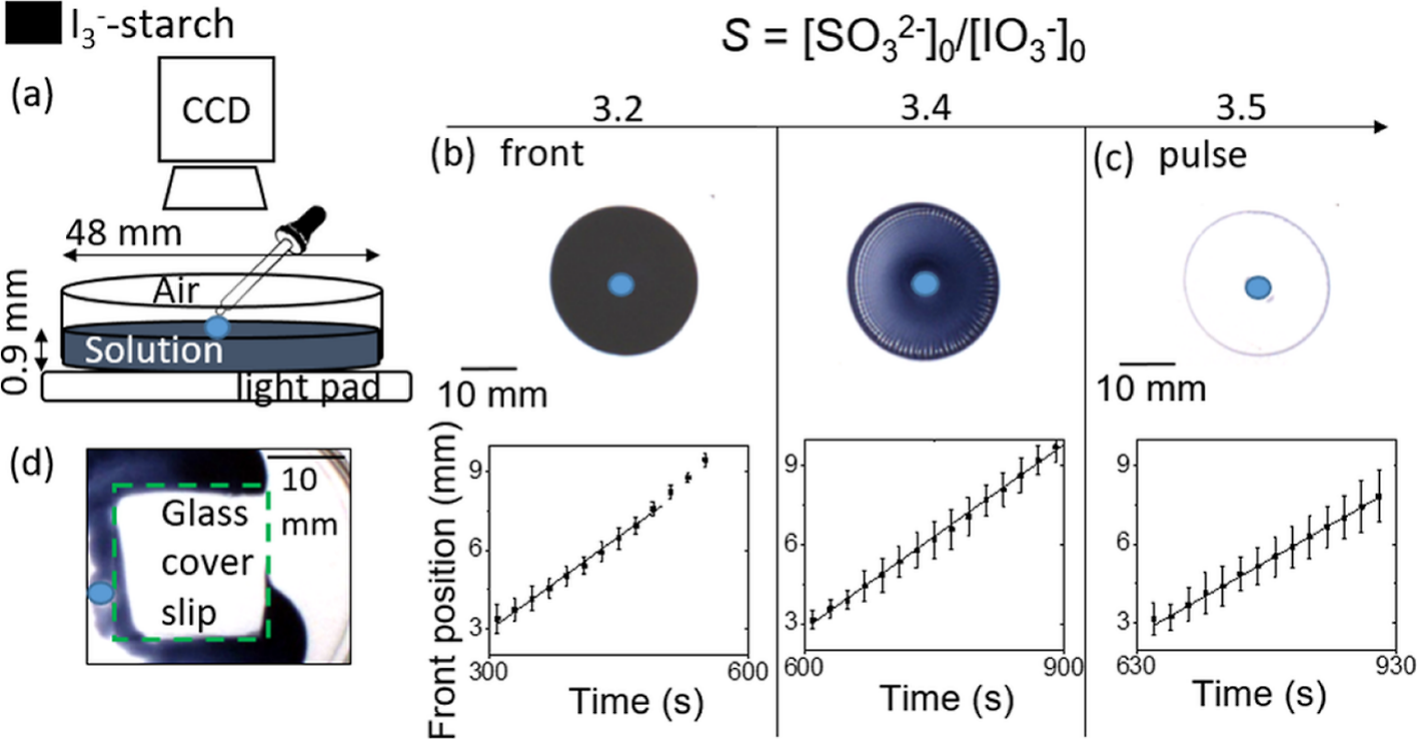
Iodine fronts and pulses as a function of the stoichiometric ratio *S* in the IS reaction in a thin layer. (a) Schematic of the experiment with a CCD camera positioned above a Petri dish containing the reaction solution at a depth of 0.9 mm and the front initiated by a drop of product solution (indicated by a blue circle). (b) Propagating iodine front with *S* = 3.2 and *S* = 3.4 and the corresponding position of front in time. (c) Pulse in iodine obtained for *S* = 3.5 and position of pulse in time. (d) Front propagation around a glass coverslip at the air–water interface with *S* = 3.4. Average position of the front and standard deviation were calculated from nine intensity profiles. The sulfite was varied in the range [SO_3_^2–^]_0_ = 0.029–0.031 mol/L, and the other fixed conditions were as follows: [KIO_3_]_0_ = 8.96 × 10^–3^ mol/L, [H_2_SO_4_]_0_ = 4.80 × 10^–3^ mol/L, and [starch] = 5.0 g/L or 0.5% (w/v). Influence of oxygen on the formation of iodine gradients.

The transition from iodine fronts to pulses in the IS reaction occurred at *S* > 3.4, where *S* = [SO_3_^2–^]_0_/[IO_3_^–^]_0_, in a thin layer (0.9 mm) in a Petri dish. The reaction was initiated by a drop of product solution (Figure 2a), and the average front velocity was determined from the slope of the position-time plot (see experimental section). For *S* = 3.2, the average front velocity was ⟨*c*⟩ = 1.43 ± 0.03 mm min^–1^, and acceleration was observed at longer times (Figure 2b). At the transition point, *S* = 3.4, the iodine concentration was reduced in the wake of the front, and the front traveled with constant velocity of ⟨*c*⟩ = 1.40 ± 0.01 mm min^–1^ (Figure 2b). With *S* = 3.5, pulses propagated with a constant velocity of ⟨*c*⟩ = 1.12 ± 0.02 mm min^–1^ (Figure 2c).

Iodine formation was not observed with values of *S* = 3.2–3.5 in the well-stirred solution as the iodine was consumed by the excess bisulfite with *S* > 3. However, with *S* = 3.2 in the simulations, an increase in the transfer rate of oxygen resulted in a transition from low- to high-iodine concentration as oxygen removed bisulfite (see Figure 1d). The appearance of iodine fronts at *S* > 3 may be attributed to the increased rate of transfer of oxygen into the thin layer of solution as a result of a larger surface area relative to the volume. In addition, with *S* = 3.4, when a glass coverslip was placed at the air–water interface, the iodine front was observed to propagate around the coverslip (Figure 2d). This result supports the postulation that transfer of oxygen is required for iodine formation under these conditions, and its influence is equivalent to a decrease in *S.* Without oxygen, the reaction finishes in a high-iodide state, and no color change is observed.

**Figure 3:**
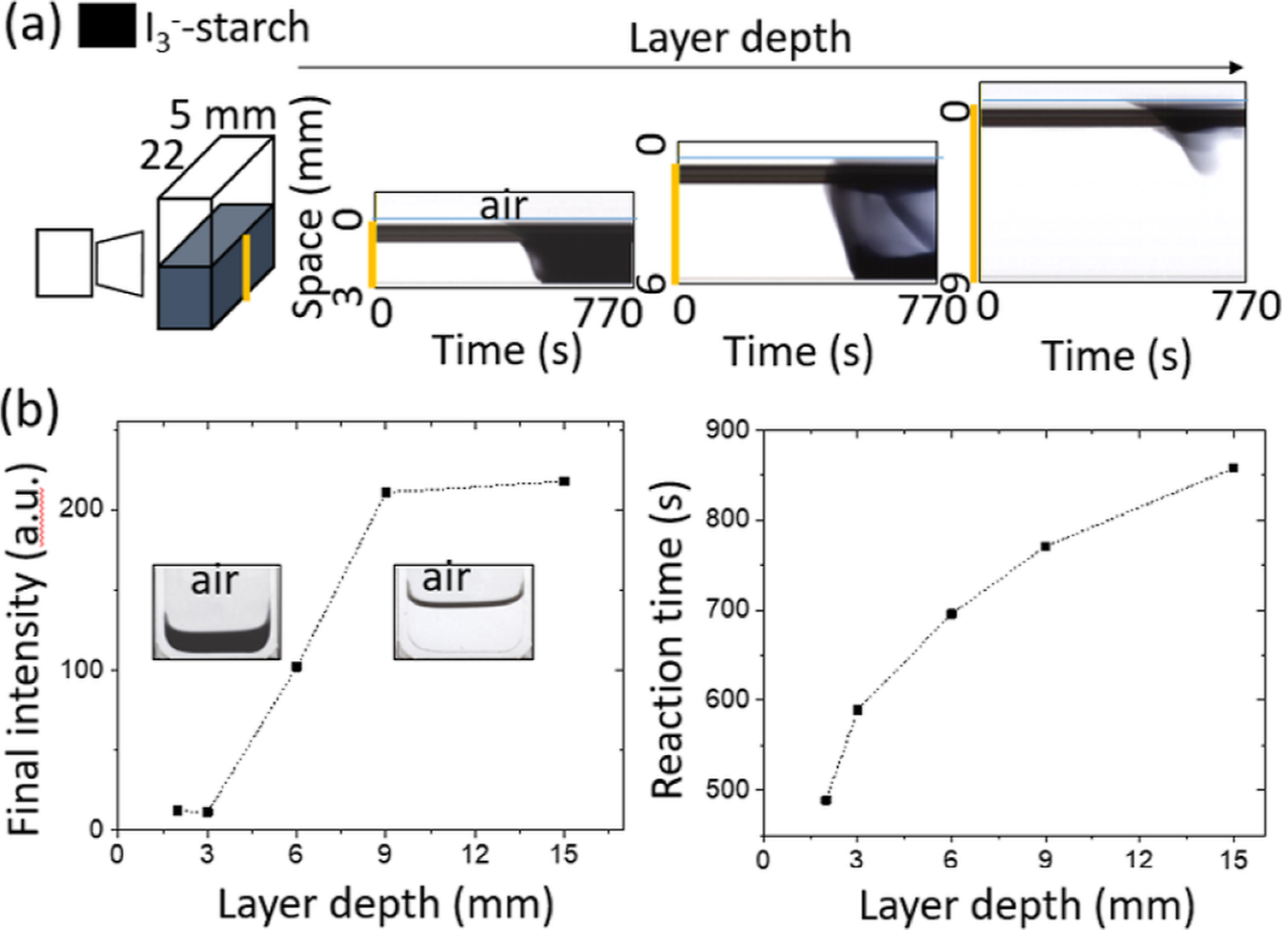
Effect of layer depth on iodine gradients formed spontaneously in the unstirred IS reaction with *S* = 3.2. (a) Schematic of side-view experiments in rectangular container with the camera position indicated and space-time plots constructed from vertical slice (yellow line) with different layer depths. (b) Average final intensity of the image (steady-state value) and reaction time (to the maximum concentration of starch-[I_3_^–^]) as a function of the layer depth. The initial concentrations were [SO_3_^2–^] = 0.029 mol/L, [KIO_3_]_0_ = 8.96 × 10^–3^ mol/L, [H_2_SO_4_]_0_ = 4.80 × 10^–3^ mol/L, and [starch] = 5.0 g/L or 0.5% (w/v).

The reaction was also performed in a thin glass container viewed from the side in order to determine the effect of oxygen on the formation of iodine gradients (Figure 3a). The value of *S* = 3.2 (*S* = [SO_3_^2–^]_0_/[IO_3_^–^]_0_) was chosen which in the well-stirred reaction resulted in no iodine formation. The container had a sufficiently large gap (5 mm) to allow transfer of oxygen, but the surface area to volume ratio was reduced compared to the Petri dish. Here, iodine developed spontaneously at the air–water interface (rather than initiated), and the amount of iodine formed depended on the layer depth. For layer depths of 3 mm, complete conversion to a high-iodine state was obtained after a period of time (space-time plot, Figure 2a). However, if the reaction was performed under nitrogen, then the final state was low-iodine as expected since *S* > 3. When the solution depth was increased to 6 mm, a lower concentration of iodine was obtained. With a solution depth of 9 mm or more, iodine was observed only in the top part of the solution, and there was a return to the low-iodine state. Hence, as the layer depth was increased, there was a switch from a high- to low-iodine final state with the formation of transient iodine gradients (Figure 3b). The reaction time, defined as the time to minimum intensity (maximum in the average concentration of starch-I_3_^–^), also increased with increasing layer depth.

**Figure 4:**
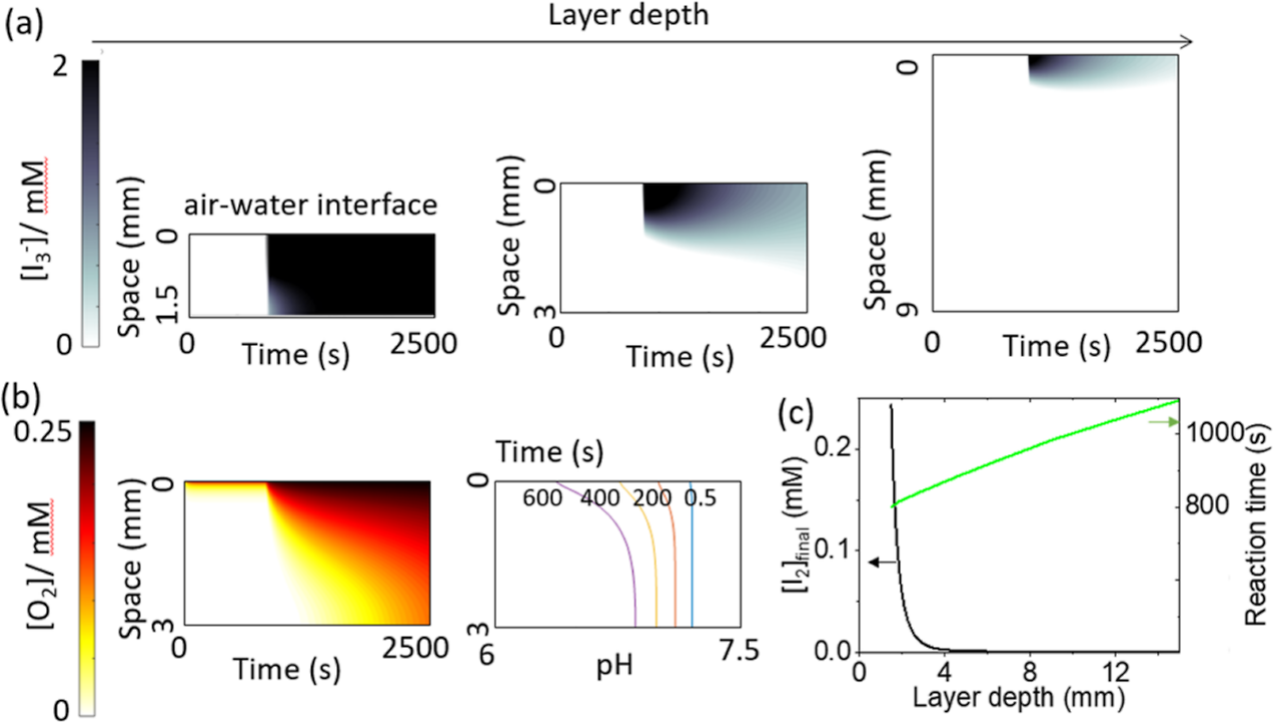
Reaction-diffusion simulations (1D) of the IS reaction with an oxygen influx at the upper boundary to illustrate the influence of oxygen on the formation of transient iodine gradients with *S* = 3.2. The initial concentrations were [IO_3_^–^] = 8.96 mM, [H^+^] = 4.8 mM, [HSO_4_^–^] = 4.8 mM, and [SO_3_^2–^] = 0.029 M. (a) Space-time plots showing formation of iodine and transition from the high-iodine state to low-iodine state as the layer depth was increased. (b) Oxygen profile in time and pH profile at different time points in a 1D system of depth 1.5 mm. (c) Final iodine concentration (*T* = 2500 s, black, lower curve) and reaction time (to minimum in [I_2_], green, upper curve) as a function of layer depth. Influence of oxygen on spontaneous iodine patterns.

The formation of transient iodine gradients with *S* = 3.2 can be explained by the effect of oxygen. To illustrate this, reaction-diffusion simulations were performed in one dimension with reactions 1–10 and a constant supply of oxygen from the upper boundary. Diffusion coefficients were considered constant, and these simulations did not take into account the potential for enhanced transport of oxygen close to the air–water interface as a result of Marangoni effect or other convective effects.^49^ Nevertheless, the space-time plots qualitatively matched the experimental results: as the layer depth was increased, there was a switch from the high-iodine state to the low-iodine state with transient iodine gradients (Figure 4a). A more pronounced effect (higher iodine for given L) is expected if convective effects are included as these will enhance oxygen levels in the upper part of the solution.

The oxygen profile in time can be observed in Figure 4b. Oxygen was rapidly consumed by reaction with bisulfite and then unable to diffuse from the interface sufficiently quickly, resulting in a gradient in oxygen. Hence, there was lower bisulfite at the interface, and iodine formed only at the top of the solution. For sufficiently deep layers, the influence of oxygen on the reaction was equivalent to having a gradient in *S,* with low *S* at the interface and high *S* at the base*.* The reaction time (to maximum [I_2_]) increased with increasing layer depth, and the final iodine concentration decreased as overall there was less oxygen available in deeper layers (Figure 4c).

**Figure 5:**
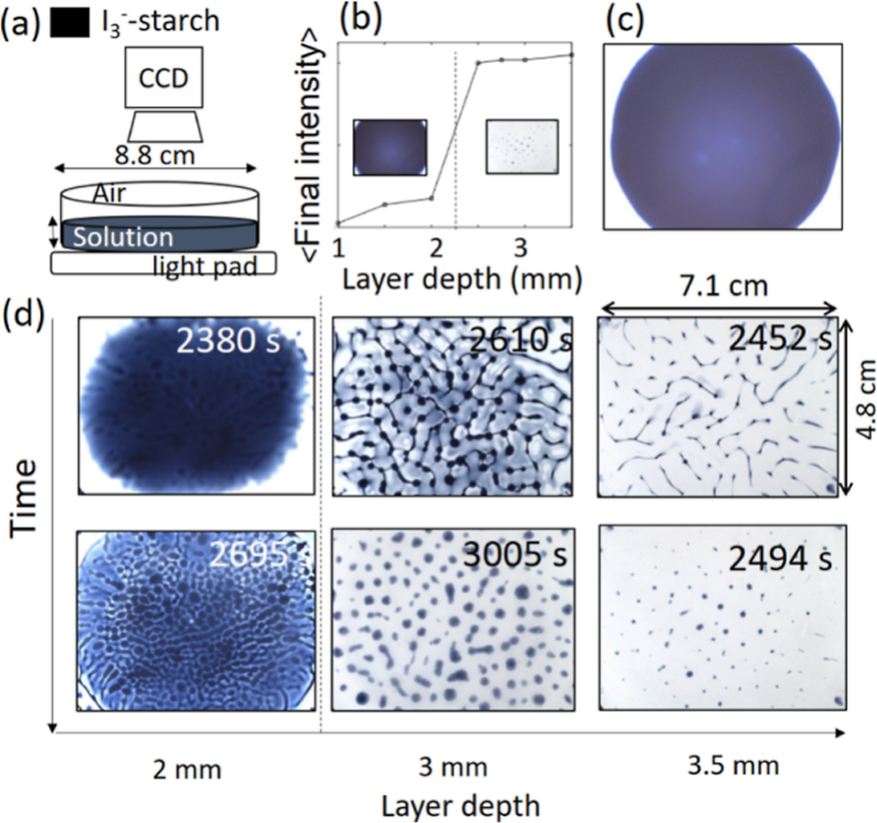
Effect of layer depth on spontaneous iodine patterns in the IS reaction in a Petri dish with *S* = 3.5. (a) Schematic of apparatus used for observation of patterns in solutions of different layer depths, L, and (b) average intensity (a.u.) of the image taken at the end of the experiment. Inset shows images of high- and low-iodine with *L* = 1 and 3 mm at 3000 s. (c) Image of iodine front at 1 mm depth. (d) Images of iodine pattern formed at different layer depths. Initial solution concentrations were [KIO_3_]_0_ = 8.96 × 10^–3^ mol/L, [Na_2_SO_3_]_0_ = 3.12 × 10^–2^ mol/L, [H_2_SO_4_]_0_ = 4.80 × 10^–3^ mol/L, and [starch] = 5.0 g/L. Field of view in images: 7.1 × 4.8 mm.

Interesting patterns were obtained in the IS reaction with *S* = 3.5 when iodine was allowed to develop spontaneously in a Petri dish for optimal layer depths. A schematic of the setup is shown in Figure 5a. Under these conditions, there was no iodine formed in the well-stirred reaction as *S* > 3; however, a transition from a high-iodine final state to a low-iodine state was obtained with increasing layer depth (Figure 5b). For sufficiently thin layers (1 mm), iodine formed typically at the center of the dish after an induction period of ∼30 min and propagated with an approximately constant velocity of 9.4 ± 0.2 mm min^–1^ (Figure 5c). When the layer depth was increased to 2 mm, an irregular front appeared after 39 min and was followed by the formation of a cellular pattern (Figure 5d). A further increase in the layer depth to 3 or 3.5 mm resulted in the formation of a network structure which evolved to a spotted pattern.

**Figure 6:**
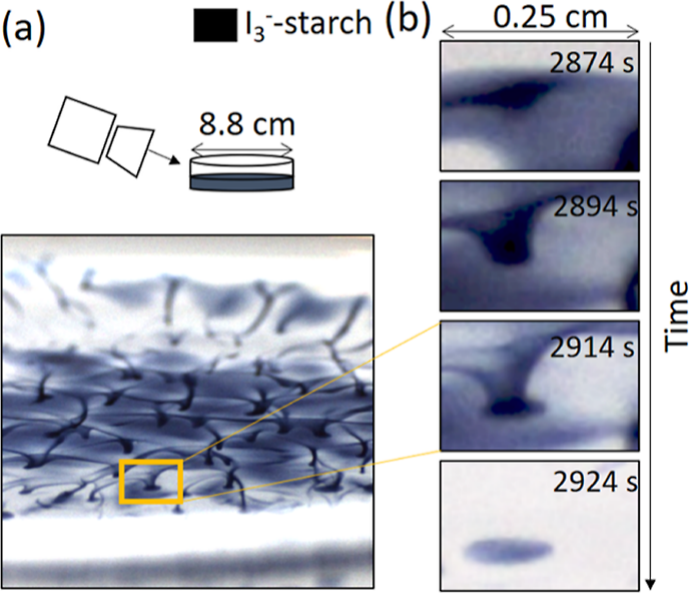
Spotted pattern formation in IS solution of 3 mm layer depth. (a) Schematic of the setup with the camera mounted at 30° to view the full length of the Petri dish and image of iodine bridges. (b) Evolution of the iodine pattern in time. The concentrations were [KIO_3_]_0_ = 8.96 × 10^–3^ mol/L, [Na_2_SO_3_]_0_ = 3.12 × 10^–2^ mol/L, [H_2_SO_4_]_0_ = 4.80 × 10^–3^ mol/L, and [starch] = 5.0 g/L (0.5%).

The most striking spotted patterns occurred between layer depths of 2.5 and 3.5 mm. A side view of the structure at an angle of 30° revealed iodine bridges that extended throughout the solution (Figure 6a). Iodine formed initially at the air–water interface in a fast moving front that left behind an iodine-rich network. At the nodes, the iodine propagated down forming quasi-2D iodine spots at the base of the dish (Figure 6b).

**Figure 7:**
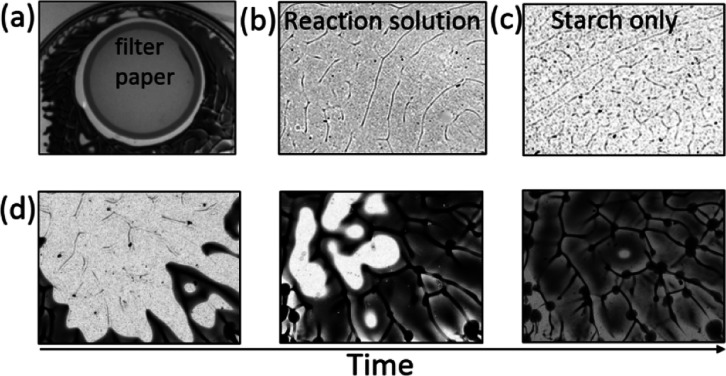
(a) Iodine patterns formed around a 47 mm diameter filter paper placed at the air–water interface in a solution of *L* = 3 mm. (b–d) Patterns observed using shadowgraph imaging. (b) IS solution in 2 mm layer prior to appearance of iodine. (c) Starch solution in 2 mm layer. (d) Evolution of network-guided iodine pattern formation in time in a layer of 3.5 mm and time interval between images of 30 s. [KIO_3_]_0_ = 8.96 mM, [Na_2_SO_3_]_0_ = 3.12 × 10^–2^ M, [H_2_SO_4_]_0_ = 4.80 mM, and [starch] = 5.0 g/L. Field of view: 2.6 × 2 cm.

The patterns shown here formed with or without a loose fitting lid, and the presence of a lid helped to stabilize the patterns from disruption by air flow. No iodine patterns were obtained if the reaction was performed under a nitrogen atmosphere, and patterns were formed around a glass cover slip placed carefully at the air–water interface. Iodine formation was also suppressed if a filter paper was placed at the interface (Figure 7a). These results suggest that oxygen plays a key role in pattern formation as it controls the amount of iodine formed.

Oxygen is required for the formation of iodine gradients; however, this does not explain the initial network pattern that is formed. Shadowgraph images of the patterns in the Petri dish revealed that the network-like structure appeared prior to the appearance of iodine (Figure 7b) and in the presence of starch alone (Figure 7c). When the formation of iodine occurred at the interface, it can be seen to propagate quickly along the pre-existing network leaving behind lines of iodine (Figure 7d). Without starch, no patterns were obtained at these layer depths, only bulk formation of iodine.

**Figure 8:**
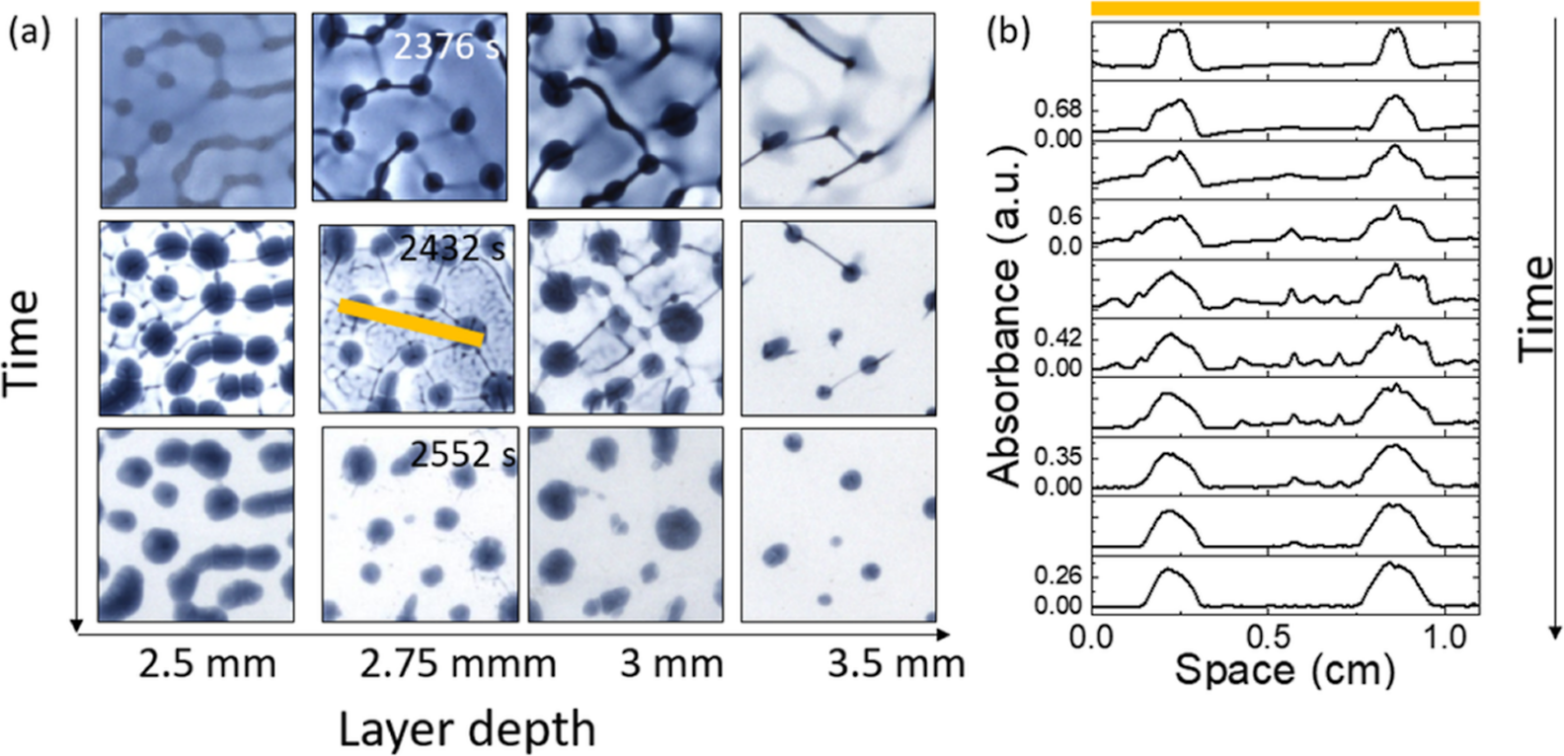
(a) Temporal evolution of the network pattern in the IS reaction in a Petri dish at different layer depths showing a coarsening of the structure in time at the air–water interface and then disappearance of iodine leading to the spotted pattern. Field of view: 1.5 × 1.5 cm. (b) Absorbance profile [log(*I*/*I*_0_)] obtained every 20 s from 2376 s along the line shown in (a) (where left edge corresponds to *x* = 0) at a layer depth of 2.75 mm.

The instabilities evolved at the interface and coarsening of the pattern was observed, resulting in an irregular substructure (Figure 8a). This suggests there is an influence of iodine on the instabilities observed at the air–water interface. Subsequently, this was followed by removal of iodine at the surface and return to a high-iodide state, leaving behind the spotted pattern at the base of the dish. The spots initially grew and then became stationary, as can be seen in the temporal evolution of the absorbance profile obtained across the line indicated in the image in Figure 8a,b. The pattern was somewhat irregular with a wavelength of the order of 3 mm. There was only a slight decrease in the pattern wavelength with the layer depth from 2.5 to 3.5 mm; however, less iodine formed and thus a sparser spot pattern prevailed. Above 3.5 mm, no iodine patterns were obtained.

## Discussion

The formation of patterns driven by coupling of hydrodynamic instabilities with chemical reaction is important across numerous fields.^1,50^ Much of the experimental work on stationary patterns in chemical systems was driven by Turing’s seminal work on a mechanism for biological pattern formation involving coupling of autocatalytic reaction with differential diffusion.^51^ Candidate systems were proposed that displayed labyrinth or spotlike patterns which were later attributed to convective effects, such as the long-lasting mosaic patterns in the methylene blue-sulfide-oxygen solution system.^52^ Similar structures can arise from different mechanisms, and often multiple effects contribute to the resultant patterns, as is the case in the IS reaction. In an open reactor with substrates continuously supplied, oscillations and spatiotemporal patterns have been observed in the IS reaction, even without the addition of an inhibitor species.^35,53^ The fast transport of acid was thought to play a role in that case.^18^ However, the closed system is not expected to show such patterns, except through the potential coupling of reaction with convective instabilities.

Oxygen plays an important role in chemoconvective pattern formation in a number of non-autocatalytic systems in which it is a key substrate.^54^ The formation of dense products can result in a buoyancy-driven instability in these cases. The influence of convective effects on fronts has been studied in the IS reaction and related IA reaction.^55,56^ Since the IS reaction is autocatalytic in iodide and protons, both iodine and pH fronts and pulses are possible.^57^ The reaction is slightly exothermic (d*H* < 0), and the isothermal density change was reported to be negative (dρ_c_ < 0), but nevertheless, front acceleration and fingering instabilities were observed in descending pH fronts in thin tubes.^56^ These were attributed to the fast diffusion of acid relative to other species which caused double-diffusive instabilities.^18^

Here, we focus on the effect of oxygen on iodine formation in the IS reaction. We have shown that the transfer of oxygen from the air could be used to control the amount of iodine that was produced, and this was important for the observation of iodine fronts and patterns for certain values of the stoichiometric ratio *S* = [SO_3_^2–^]/[IO_3_^–^]. In the well-stirred reaction when *S* < 3, iodine was formed, whereas for *S* > 3, iodide was formed in excess. For *S* = 3.2, kinetic simulations of the well-stirred reaction demonstrated that there was a switch from a low-iodine to high-iodine state as the rate of transport of oxygen was increased. Oxygen removed sulfite in an alternative reaction channel, thus having the same effect as decreasing *S* and favoring the formation of iodine.

The formation of iodine fronts or pulses in the IA reaction depends on the stoichiometric ratio *R* of the reductant, arsenous acid, to iodate, with fronts forming at *R* < 3 and pulses at *R* ∼ 3.^46,47^ We observed iodine fronts and pulses in the IS reaction propagating with a constant velocity of 1–1.4 mm min^–1^ in a Petri dish open to air with layer depths of *L* = 0.9 mm. The switch from fronts to pulses occurred at a higher than expected stoichiometric ratio of *S* = 3.4. The observation of fronts at *S* > 3 was attributed to the enhanced transport of oxygen into the thin layer, removing sulfite and hence resulting in an increase in iodine.

The effect of oxygen on the reaction was also examined in unstirred solutions in a thin container viewed from the side with *S* = 3.2. The layer depth influenced the reaction outcome: for shallow layers, the final state was iodine, whereas in deeper layers, insufficient oxygen could penetrate, so the final state was iodide. Transient gradients in iodine also formed for sufficiently deep layers. The 1D model was used to show how oxygen drives the formation of gradients in iodine. This spatial separation of iodine states facilitated pattern formation when iodine was allowed to evolve spontaneously in a thin layer in a Petri dish with *S* = 3.5. Under these conditions, formation of iodine is not expected; however, for optimal layer depths, short-lived surface iodine patterns evolved to give a stationary quasi-2D spotted pattern at the base of the dish. Thus, the gradients in oxygen and hence iodine played a key role in the evolution of patterns.

In the IA reaction, acceleration of fronts in horizontally propagating fronts in Hele-Shaw cells or a Petri dish with an open air interface were attributed to a Marangoni instability driven by the formation of iodine.^48,55^ Pulses propagated with a constant velocity of the order of 1 mm min^–1^, whereas acceleration of the fronts was observed at *R* < 2.8 during the initial and late phases of the reaction. During the intermediate phase, the velocity was constant and of the order of 8 mm min^–1^ for *R* = 2.6 as presumably, evaporation of iodine matched its production. Iodine lowers surface tension, resulting in a large solutal Marangoni number and fluid flow that enhances the front velocity. It seems likely that iodine surface activity contributes to the spontaneous pattern development observed in the IS reaction in a Petri dish; front velocities of ∼ 9 mm min^–1^ were obtained in layers of *L* = 1 mm, and coarsening of the network-like patterns was observed at the air–water interface with *L* = 2–3 mm.

However, the network-like pattern was present before the appearance of iodine at the air–water interface.^58^ Similar spatial structures were observed in photochemical reactions at liquid/air interfaces or even nonreactive processes in a Petri dish.^58,59^ In some cases, these were attributed to a thermal Marangoni instability involving evaporative cooling.^32,60^ The instability resulted in a cellular prepattern, and the chemicals traced the structure. The prepatterns observed here occurred in starch solution alone, and starch is also known to be surface-active.^61^ Therefore, it is possible that the initial pattern was driven by Marangoni effects, and the coupling of the initial Marangoni instability with oxygen-driven iodine gradients is responsible for spotted pattern formation in this clock reaction system. Starch and the starch-tri-iodide complex also influence the solution viscosity;^62^ further investigation is required for a complete understanding of all factors involved in these striking 3D patterns.

## Conclusions

We have shown that oxygen can be used to control the switch from a low- to high-iodine state in the IS reaction, with oxygen-rich solution favoring iodine formation as a result of bisulfite removal. Solutions that are open to the air develop concentration gradients such that iodine forms first at the interface. At optimal layer depths, the coupling of the iodine gradient with Marangoni instability resulted in a striking network pattern at the surface that evolved to a spotted pattern at the base of the dish. This work illustrates how chemical gradients in species such as oxygen can drive rich dynamical pattern formation in autocatalytic reaction systems that show simple clock behavior or propagating fronts. There are other autocatalytic systems that exploit sulfite, for example, reactions with bromate and formaldehyde, and these processes have been coupled with material formation for time-lapse gelation and particle formation. Oxygen may play an important role in the spatiotemporal control formation of the material structure in these systems.
